# Dose-dependent progression of multiple low-dose streptozotocin-induced diabetes in mice

**DOI:** 10.1152/physiolgenomics.00032.2023

**Published:** 2023-07-17

**Authors:** Brandon M. Bauer, Supriyo Bhattacharya, Elizabeth Bloom-Saldana, Jose M. Irimia-Dominguez, Patrick T. Fueger

**Affiliations:** ^1^Department of Molecular & Cellular Endocrinology, Arthur Riggs Diabetes & Metabolism Research Institute, City of Hope, Duarte, California, United States; ^2^Irell & Manella Graduate School of Biological Science, Beckman Research Institute, City of Hope, Duarte, California, United States; ^3^Integrative Genomics Core, Beckman Research Institute, City of Hope, Duarte, California, United States; ^4^Comprehensive Metabolic Phenotyping Core, Beckman Research Institute, City of Hope, Duarte, California, United States

**Keywords:** beta cell mass, hyperglycemic clamp, insulin secretion, transcriptomics

## Abstract

This study investigated the effects of different multiple low doses of streptozotocin (STZ), namely 35 and 55 mg/kg, on the onset and progression of diabetes in mice. Both doses are commonly used in research, and although both induced a loss of beta cell mass, they had distinct effects on whole glucose tolerance, beta cell function, and gene transcription. Mice treated with 55 mg/kg became rapidly glucose intolerant, whereas those treated with 35 mg/kg had a slower onset and remained glucose tolerant for up to a week before becoming equally glucose intolerant as the 55 mg/kg group. Beta cell mass loss was similar between the two groups, but the 35 mg/kg-treated mice had improved glucose-stimulated insulin secretion in gold-standard hyperglycemic clamp studies. Transcriptomic analysis revealed that the 55 mg/kg dose caused disruptions in nearly five times as many genes as the 35 mg/kg dose in isolated pancreatic islets. Pathways that were downregulated in both doses were more downregulated in the 55 mg/kg-treated mice, whereas pathways that were upregulated in both doses were more upregulated in the 35 mg/kg-treated mice. Moreover, we observed a differential downregulation in the 55 mg/kg-treated islets of beta cell characteristic pathways, such as exocytosis or hormone secretion. On the other hand, apoptosis was differentially upregulated in 35 mg/kg-treated islets, suggesting different transcriptional mechanisms in the onset of STZ-induced damage in the islets. This study demonstrates that the two STZ doses induce distinctly mechanistic progressions for the loss of functional beta cell mass.

## Graphical abstract

**Figure:**
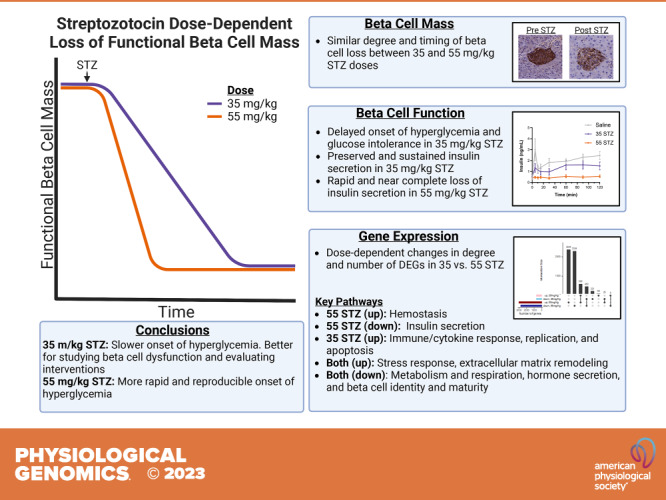

## INTRODUCTION

Type 1 diabetes mellitus (T1D) results from the autoimmune-mediated destruction of the insulin-producing beta cells in the islets of Langerhans in the pancreas. Without a cure for T1D, the field continues to rely on experimental model systems to identify potential therapeutics. Two of the main model systems used are the nonobese diabetic mouse (NOD) and beta cell destruction models using chemical agents such as alloxan and streptozotocin (STZ).

STZ is an alkylating agent that is selectively cytotoxic to beta cells (1–3). It is a common experimental tool to study the loss of beta cell mass in rodents as well as beta cell replacement strategies (4). STZ induces DNA damage, oxidative stress, and apoptosis, leading to chronic pancreatic islet inflammation, insulitis, and insulin deficiency, which resembles features of human T1D. However, the complete mechanisms governing beta cell mass loss in STZ-induced models of diabetes remain incompletely understood (2). Whereas a single injection of a high dose (≥150 mg/kg) of STZ can induce a near-complete necrotic ablation of beta cells within 24 h and overt hyperglycemia within 48 h, multiple administrations of a lower dose of STZ results in a more gradual loss of beta cell mass and delayed hyperglycemia, a phenotype not directly attributable to the drug cytotoxicity (4). Therefore, multiple low-dose administration of STZ is often preferred as a model of human disease since it induces beta cell changes that model diabetes progression such as immune infiltration and beta cell dysfunction (5, 6). In addition, low-dose STZ has been used to study beta cell regeneration and repair (7–9). However, there remains little uniformity in the field when it comes to selecting a low STZ dose.

The original work to introduce the multiple-dose technique used 40 mg/kg (10), but doses as low as 30 mg/kg or as high as 55 mg/kg are commonly used. We sought to compare the impact of a multiple low dose (55 mg/kg) to a multiple very low dose (35 mg/kg) administration of STZ. Our goal was to better understand how the STZ concentration alters the metabolic, morphological, functional, and transcriptomic progression of STZ-induced rodent beta cell dysfunction as a model of T1D.

We identified key differences in disease progression between a 35 and 55 mg/kg multiple low dose of STZ. While 55 mg/kg-treated animals became glucose intolerant within 3 days following STZ administration, 35 mg/kg-treated animals remained glucose tolerant for several days longer than 55 mg/kg-treated animals despite an equivalent loss of beta cell mass. Using hyperglycemic clamps, the gold standard for quantifying insulin secretion in vivo, we confirmed that 35 mg/kg-treated mice partially retained glucose-stimulated insulin secretion for an extended period following treatment compared with that of 55 mg/kg-treated mice. There were also significant differences in gene expression between the two treatment groups, including dose-dependent shifts in differentially expressed genes and pathways as well as a subset of genes altered in opposite directions. This work clarifies the impact of STZ concentration and can help researchers to select an appropriate dose for their experiments to properly model disease progression and improve intervention models to retain or restore beta cell mass in diabetes.

## METHODS

### Animal Studies

All animals were maintained in accordance with City of Hope Institutional Animal Care and Use Committee approved protocols (No. 16047) in accordance with the Guide for the Care and Use of Laboratory Animals (11). Only male C57BL/6J mice were used due to the varying effectiveness of STZ in female mice. All mice were housed in individually ventilated cages with 3–5 mice/cage. Animals were maintained on 12:12-h light:dark cycles with ad libitum access to water and standard rodent chow diet.

STZ (Sigma-Aldrich S0130-1G; St. Louis, MO) was administered for 5 consecutive days by intraperitoneal injections of STZ dissolved right before the injection in saline at either 35 or 55 mg/kg body weight. Saline was administered to 34 mice as a vehicle control, 40 mice received 35 mg/kg STZ, and 38 mice received 55 mg/kg STZ. Equivalent volumes of saline were used in control animals. Glucose tolerance tests were performed on 10- to 12-wk-old male mice following a 5-h fast; glucose (1.5 g/kg) was delivered by intraperitoneal injections. Blood glucose measurements were made using an AlphaTRAK 2 (Abbott Laboratories; Abbott Park, IL) glucometer from a tail nick blood sample.

### Quantification of Beta Cell Mass

Pancreata were removed from mice following CO_2_ asphyxiation, and tissues were fixed in 10% formalin overnight. Pancreas slices were processed into formalin blocks with assistance from the Solid Tumor Pathology Core at City of Hope, and 6-µm section slides were made from the paraffin blocks. Paraffin removal was performed by immersion in xylene, and slide rehydration was achieved by serial washes in graded ethanol solutions. Antigen retrieval was performed by boiling slides in pH 6.0 citrate buffer (H3300; Vector Laboratories; Newark, CA) for 10 min. Slides were subsequently allowed to cool to room temperature for 30 min. Slides were blocked with serum-free protein block (Dako X0909; Agilent Technologies; Santa Clara, CA). Insulin was labeled with primary guinea pig anti-insulin antibody (1:500; ab195956; Abcam; Waltham, MA) at 4°C overnight and detected with secondary biotinylated goat anti-guinea pig antibody (Vector Laboratories; BA7000, 1:500) followed by Vectastain Elite ABC HRP detection kit (Vector Laboratories; PK-6100) and diaminobenzidine (DAB) (K346811-2; Agilent Technologies). Slides were counterstained with Mayer’s hematoxylin (TA-125-MH; Epredia; Kalamazoo, MI).

Slides were imaged using a NanoZoomer 2.0HT (Hamamatsu Photonics; Shizuoka, Japan) slide scanner and analyzed using QuPath (V.0.3.2). The pixel identification feature of QuPath (12, 13) was used to automatically identify and quantify either total pancreas area or insulin-positive area in a blinded manner. All images were checked for the presence of positive identification of artifact and were manually corrected when necessary.

### Hyperglycemic Clamps

Surgical implantation of carotid artery and jugular vein catheters and hyperglycemic clamp procedures to assess in vivo beta cell function was performed by the Comprehensive Metabolic Phenotyping Core at City of Hope according to the techniques previously described (14–16). In brief, mice were anesthetized with isoflurane and surgery was conducted using aseptic techniques. An arterial catheter was inserted through an incision, terminating ∼9.5 mm into the carotid artery lumen, and was secured by proximal and distal ligatures. A second catheter was inserted into the right jugular vein terminating ∼10 mm into the vessels lumen and secured with proximal and distal ligatures. The free ends of each catheter were tunneled subcutaneously toward the back of the mouse, exteriorized through a small incision in the interscapular region, and connected to the mouse antenna for sampling access (MASA; made in-house) device. The MASA device was then secured with suture, making blood sampling/infusion ports easily accessible from the interscapular region of the mouse. Animals were provided analgesia and fluids and monitored postoperatively.

After 4 days of recovery, mice that returned to within 10% of presurgical body weight were attached to a two-way swivel (Instech, Plymouth Meeting, PA), allowing animals to remain conscious and freely moving throughout the procedure. Animals received a primed infusion (50 mg/kg/min for 2 min) followed by variable infusion of 50% dextrose and continuous infusion of saline-washed erythrocytes throughout the experiment, to achieve hyperglycemia (target: 270 mg/dL or 18 mM) and to maintain a constant hematocrit, respectively. Arterial blood glucose was monitored during the clamp every 10 min (∼5 µL whole blood). Blood samples (∼100 µL each) were collected at −15, −5, 5, 10, 15, 30, 60, 90, and 120 min to measure blood insulin (Sensitive Rat Insulin Kit, Cat. No. SRI-13K; MilliporeSigma; Burlington, MA) following the manufacturer’s instructions. Mice were euthanized at the conclusion of the study, and the pancreas was collected for histology.

### Islet Isolation

Islets were isolated using methods previously described by Stull et al. (17) with freshly prepared CIzyme RI collagenase and protease solution (VitaCyte 005–1030; Indianapolis, IN). After isolation, healthy islets were manually picked and cultured overnight at 37°C with 5% CO_2_ in RPMI supplemented with 10% FBS, 5 mM HEPES (Fisher Scientific, BP310-500), and penicillin/streptomycin (50 units/mL and 50 µg/mL, respectively; Gibco/Thermo Fisher Scientific, 15-140-122; Waltham, MA).

### RNA Sequencing

Approximately 75–100 healthy islets per mouse were picked by hand, washed in cold PBS, and then resuspended in 350 µL of RLT with 1% beta-mercaptoethanol (BME). RNA was isolated using RNeasy Micro Kits (Qiagen 7404; Hilden, Germany) according to the manufacturer’s instructions and DNA was digested by treatment with RNAse-Free DNAse Set (Qiagen 79254).

RNA sequencing libraries were prepared using 150 ng total RNA with Kapa RNA mRNA HyperPrep Kit (Kapa Biosystems, Cat. No. KR1352; Cape Town, South Africa) to enrich for mRNA according to the manufacturer’s protocol with a final PCR cycle of 12 times. The RNA fragment time was 8 min at 94°C; 1.0X beads purification was used for the final sequencing library purification. The final libraries were validated with the Agilent Bioanalyzer DNA High Sensitivity Kit and quantified with Qubit. Sequencing was performed on Illumina NovaSeq 6000 by using S4 kit v1.5 with the paired end mode of 2 × 101 cycle. Real-time analysis (RTA) v3.4.4 software was used for base calling. The number of biological replicates was as follows: saline control: 6; 35 mg/kg STZ: 7; 55 mg/kg STZ: 8. The RNA sequencing yielded ∼60–70 million paired-end reads per sample.

### Data Analysis

For RNA-seq data, raw fastq reads were preprocessed to remove adapters using Trim Galore (18, 19), followed by removal of low-quality bases and mismatched base pairs using fastp (20). The cleaned reads were aligned to the reference genome (GRCm39) using STAR (21). The gene counts were obtained using HTSeq (22) according to GENCODE genome annotations, followed by conversion to counts per million (CPM). A minimum CPM threshold was determined based on median library size. Only genes that had read counts beyond the minimum CPM threshold in at least 70% of the samples were retained. DESeq2 (23) was used for normalizing the gene counts and differential gene expression (DGE) analysis. *P* values of DGEs were estimated using Student’s *t* distribution and adjusted to control false discovery according to the Benjamini–Hochberg method (24). Genes with adjusted *P* value < 0.1 were considered to be significantly differentially expressed. Geneset enrichment analysis (GSEA) was performed using clusterProfiler (25) using Gene Ontology—Biological Process (GO-BP) terms (26, 27). Protein interaction analysis was performed using STRING (28). The volcano plots, upset plot, and heatmaps were created using EnhancedVolcano (29), UpSetR (30) and ComplexHeatmap packages (31), respectively. The analysis pipeline was implemented in R version 4.2 (32).

All other data are presented as means ± SE. Adjusted area-under-the-curve (AUC) was calculated using the trapezoid method. Student’s *t* test (unpaired, two-tailed unless otherwise stated) or ANOVA (with Bonferroni post hoc tests) was performed using GraphPad Prism software (La Jolla, CA) to detect statistical differences. *P* < 0.05 was considered statistically significant.

## RESULTS

### Divergent Progression of Metabolic Dysfunction Is STZ Dose Dependent

**Figure 1.**
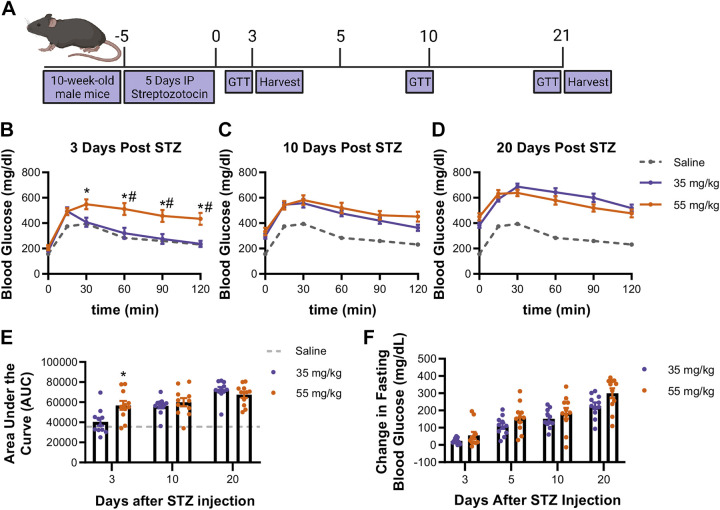
Impact of 35 mg/kg vs. 55 mg/kg dose STZ on glucose tolerance and fasting blood glucose. *A*: mice were treated for 5 consecutive days with either 35 mg/kg or 55 mg/kg STZ. *B*: glucose tolerance was assessed by an IP-GTT (1.5 g/kg glucose) at 3 days following the final day of STZ. Additional GTTs were performed at 10 days (*C*) and 20 days (*D*) following the final day of STZ. *E*: the area under the curve (AUC) of the GTTs was found at 3 days and similar at 10 and 20 days post-STZ. *F*: fasting blood glucose on *days 3*, *5*, *10*, *20* (2-way ANOVA, *P < 0.05 vs. control; #*P* < 0.05 vs. 35 mg/kg STZ, *n* = 6 or 7 mice, means ± SE). IP-GTT, intraperitoneal glucose-tolerance test; STZ, streptozotocin. Image created with a licensed version of BioRender.com.

To better define the impact of streptozotocin dose on the progression of functional beta cell mass loss in mice, 10-wk-old male mice were administered intraperitoneal injections of STZ for 5 consecutive days at either a low dose of 55 mg/kg or a very low dose of 35 mg/kg. Before the STZ administration (control) and on the 3rd, 10th, and 20th days following the final dose of STZ, glucose tolerance tests were performed by challenging 5-h fasted mice with 1.5 g/kg glucose by intraperitoneal injection (Fig. 1*A*).

At 3 days after STZ treatment, there were significant differences in glucose tolerance between the 35 and 55 mg/kg-treated mice (Fig. 1*B*). While the 35 mg/kg-treated mice resembled control (i.e., saline-treated) animals, 55 mg/kg-treated mice were significantly less glucose tolerant than both control and 35 mg/kg-treated mice. The difference in glucose tolerance, however, was not retained, as 35 mg/kg STZ-treated mice became comparably glucose intolerant by the 10th day following treatment, and through 20 days following STZ administration (Fig. 1, *C*–*E*). Five-hour fasting blood glucose continued to increase throughout the 3 wk following STZ administration in both STZ treatment groups (Fig. 1*F*). There remained a slight, albeit not statistically significant, trend in decreased fasting blood glucose between the very-low- and low-dose STZ-treated animals.

### Beta Cell Mass Loss Progresses Similarly under Both STZ Regimens

**Figure 2.**
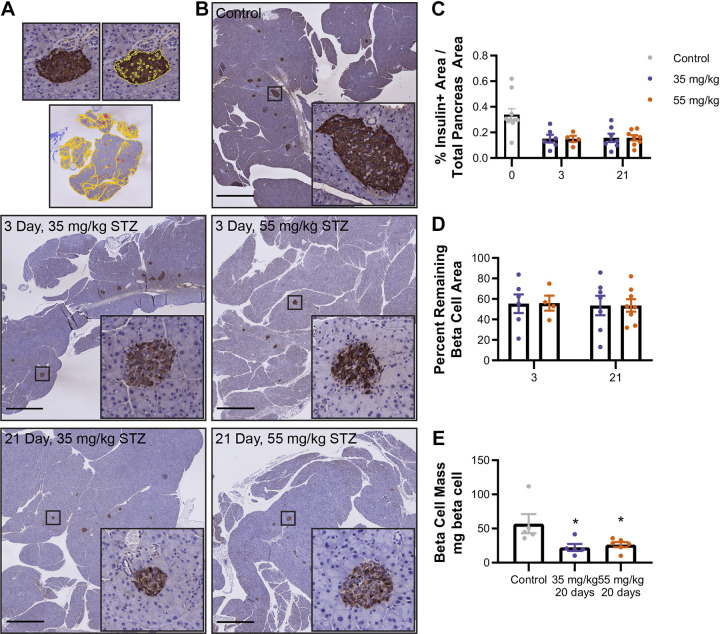
STZ-induced loss of beta cell mass is comparable between 35 mg/kg and 55 mg/kg STZ. *A*: model of insulin-positive area detection by QuPath. *B*: representative images of pancreas sections from STZ-treated mice labeled for insulin by IHC and counterstained with hematoxylin. Shown are whole pancreas images (scale bar = 1 mm) or individual islets (×20 magnification). Pixel identification in QuPath was used to identify whole pancreas area and insulin-positive tissue area. *C*: there was a significant decrease in insulin + area after STZ without any differences between the two doses. *D*: overall, there was about a 40% decrease in beta cell mass following treatment with STZ for both doses. *E*: beta cell mass (beta cell area × pancreas weight) was decreased compared with controls but similar between STZ doses. (2-way ANOVA, *P < 0.05 vs. control; *n* = 6 or 7 mice, means ± SE). IHC, immunohistochemistry; STZ, streptozotocin.

To establish the impact of STZ dose on beta cell mass, we quantified pancreatic beta cell area via immunohistochemical labeling of insulin-positive cells (Fig. 2*A*). By the 3rd day following STZ treatment, beta cell area had already declined by greater than 40% in both the 35 mg/kg- and 55 mg/kg-treated mice (Fig. 2, *B*–*D*). Interestingly, after the initial loss of beta cell mass within the first several days after STZ treatment, beta cell area did not continue to decline at the same rate despite the progressive worsening in glucose tolerance. Both beta cell area (Fig. 2, *C* and *D*) and mass (Fig. 2*E*) remained similar between the two treatment groups, which suggests loss of beta cell mass, as judged by imaging of insulin-positive cells, alone was not the primary factor impacting disease progression after the initial insult induced by STZ. We posited there might instead be differences in beta cell function, rather than beta cell mass, between the two treatment groups.

### Hyperglycemic Clamps Elucidate Dose-Dependent Loss of Insulin Secretion

**Figure 3.**
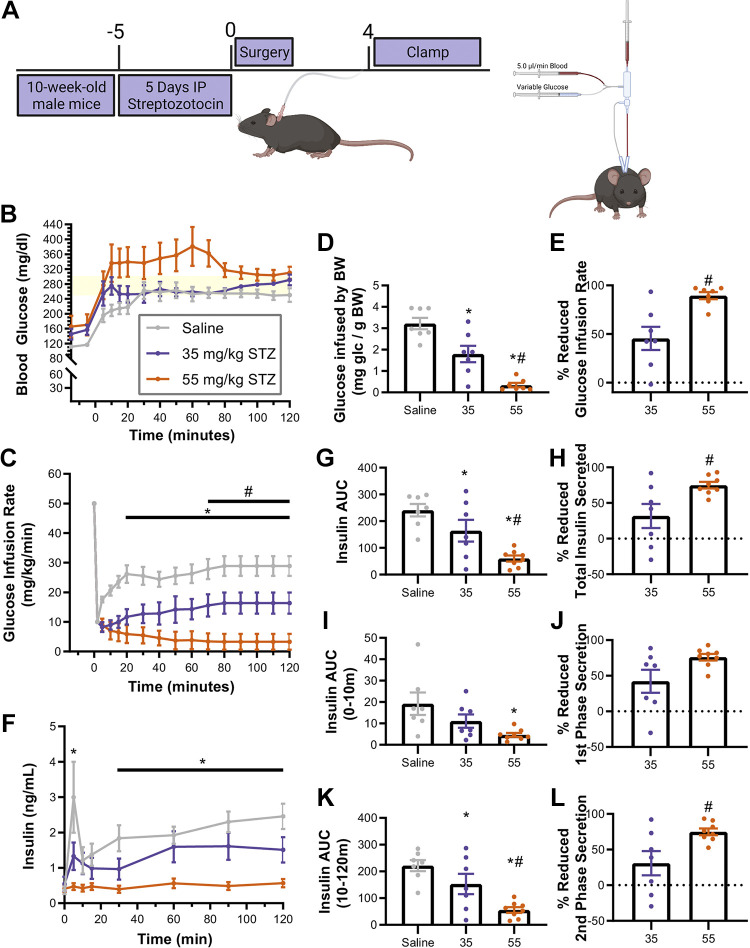
STZ (35 mg/kg) has prolonged period of glucose-stimulated insulin secretion before complete loss of beta cell function. *A*: timeline demonstrating the administration of STZ followed by surgical implantation of catheters and hyperglycemic clamp procedure. *B*: mice were maintained at a constant hyperglycemic target between 250 and 300 mg/kg for 2 h. *C*: glucose was infused at a variable rate (mg/kg/min) to maintain hyperglycemia. Total glucose infused (mg glucose/g body weight) (*D*) and percent reduced glucose infusion (*E*) compared with CON were lower in 35 and 55 mg/kg STZ. *F*: circulating insulin was assessed at 5, 10, 15, 30, 60, 90, and 120 min. *G* and *H*: area under the insulin curve was lower in 35 and 55 mg/kg STZ than CON and lower in 55 than 35 mg/kg STZ. Insulin secretion was lower in 55 mg/kg during first phase (0–10 min; *I* and *J*) and lower in 55 and 35 mg/kg STZ (*K* and *L*) than CON during second phase (10–120 min) (2-way ANOVA, *P < 0.05 vs. saline; #*P* < 0.05 vs. 35 mg/kg STZ, *n* = 6 or 7 mice, means ± SE). CON, control; STZ, streptozotocin. Image created with a licensed version of BioRender.com.

Given the improved glucose tolerance at 3 days post-STZ in the 35 mg/kg group compared with the 55 mg/kg group, despite similar remaining beta cell area, we hypothesized that the 35 mg/kg-treated mice retained beta cell function in the remaining beta cells. To determine how beta cell function was impacted by STZ dose, we performed hyperglycemic clamps, considered the gold standard methodology for quantifying in vivo insulin secretion, in live, conscious animals (33). We, therefore, treated mice with streptozotocin, or saline as control, for 5 days as previously described. On the 5th day of injection (*day 0* of posttreatment), we surgically implanted catheters, and after allowing the animals to recover from surgery, hyperglycemic clamp experiments were performed on the 4th day after surgery recovery to coincide with the period of preserved glucose tolerance we observed in the 35 mg/kg-treated mice (Fig. 3*A*).

The goal of the clamp is to achieve a stable blood glucose within the target range (250–300 mg/dL), reaching steady state in glucose infusion rate (GIR). Coinciding with the results reported in Fig. 1, the STZ-treated animals from both groups had higher basal/fasting blood glucose compared with the control animals, suggesting that the surgical procedure did not interfere with the STZ-induced glucose intolerance. All animals achieved a steady state by 10 min at which point they maintained stable blood glucose throughout the procedure (Fig. 3*B*).

GIR was decreased in both STZ-treated groups compared with the saline-treated control group after 10 min through the end of the clamp. In the mice treated with 55 mg/kg STZ, GIR values were also decreased compared with the 35 mg/kg group after 50 min (Fig. 3*C*). Overall, total glucose infused was significantly lower in 35 mg/kg-treated animals than saline-treated animals, and both parameters were even lower in 55 mg/kg-treated mice (Fig. 3*D*). The 35 mg/kg-treated mice had 45.4 ± 11.9% overall reduction in GIR and the 55 mg/kg-treated mice had 89.5 ± 3.7% reduction (Fig. 3*E*).

Saline-treated animals secreted the highest levels of insulin throughout the procedure (Fig. 3*F*). Although 35 mg/kg-treated animals tended to secrete less insulin, the only individual timepoint that was significantly lower was at 2 min. Overall insulin AUC (Fig. 3*G*) was different between all groups; however, while 55 mg/kg-treated mice had a 75.0 ± 13% loss of insulin, 35 mg/kg-treated had only a 33.8% loss (Fig. 3*H*).

We also analyzed the loss of insulin AUC in the first phase (0–10 min, Fig. 3, *I* and *J*) versus the second phase (10–120 min, Fig. 3, *K* and *L*) of insulin release. In 35 mg/kg-treated animals, although the peak insulin secretion at 2 min was lower than saline-treated controls, first-phase insulin AUC was not significantly lower than saline-treated animals (Fig. 3*I*). On the other hand, in 55 mg/kg-treated animals, insulin levels were not increased from baseline and were significantly lower than saline and 35 mg/kg groups throughout most of the clamp period (Fig. 3, *F*–*L*). Moreover, in 55 mg/kg animals, first-phase insulin release was nearly completely abolished (Fig. 3, *I* and *J*) and second phase was robustly reduced (Fig. 3, *K* and *L*). Overall, these data suggest that glucose-stimulated insulin secretion was at least partially preserved at 4 days in 35 mg/kg STZ-treated animals, and 55 mg/kg STZ-treated animals had a more rapid loss of both first- and second-phase insulin secretion compared with 35 mg/kg STZ-treated animals.

### Transcriptomic Changes Associated with STZ Dosage

**Figure 4.**
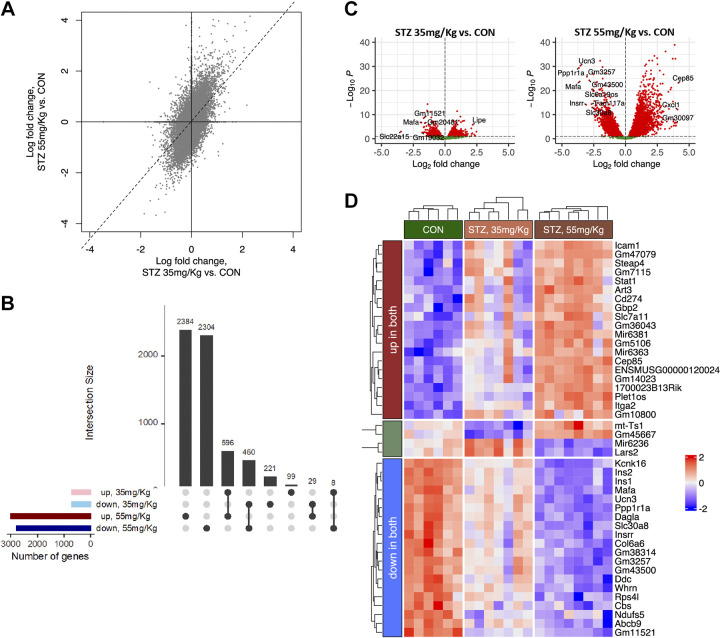
Impact of STZ dose on gene expression. *A*: correlation among log fold-changes of gene expressions between the two STZ doses. Dotted line represents the linear regression fit, along with the corresponding r2 highlighted in the plot. *B*: upset plot showing the overlap of upregulated and downregulated genes (adjusted *P* value of differential expression < 0.1) between the two STZ groups. *P* value of overlap: 6.3 × 10^−259^ (Fisher’s exact test). *C*: volcano plots of expression log fold-changes vs. statistical significance for the two STZ groups. The top up and down genes are labeled in each plot. *D*: heatmap highlighting the expressions of genes that show similar and opposite direction of change between the two STZ groups. Each column represents one mouse sample. Genes are first filtered by statistical significance (adjusted *P* value < 0.1) and those showing log fold-change greater than ±1 are retained. Top 10 genes in up/down category are shown, while only 4 genes were found to change expression in the opposite direction between the 35 and 55 mg/kg STZ groups. Each row is individually scaled between ±2. CON, control; STZ, streptozotocin.

Given that the 55 mg/kg and 35 mg/kg STZ-treated mice had differences in beta cell function shortly after STZ, we sought to characterize transcriptional changes that might be driving these differences in beta cell function. We isolated primary mouse islets on the 3rd day following treatment with STZ or saline, allowed the islets 12 h to recover from the isolation process, and then performed bulk RNA sequencing to identify differentially expressed genes (adjusted *P* < 0.1) in STZ-treated mice compared with saline-treated control mice (Supplemental Table S1: https://doi.org/10.5281/zenodo.8045137). The majority of differentially expressed genes in 35 mg/kg-treated mice were similarly differentially expressed in 55 mg/kg-treated animals. Mice treated with 55 mg/kg STZ however had substantially more differentially expressed genes. We identified 596 upregulated and 460 downregulated genes in the 35 mg/kg-treated animals. In 55 mg/kg-treated animals, there were nearly five times more differentially expressed genes with 2,384 genes upregulated and 2,304 genes downregulated (Fig. 4, *A* and *B*, and Supplemental Table S1).

Similar to other studies examining the impact of STZ on gene expression (34), we identified increased expression of genes associated with cytokine response and inflammation (e.g., *Gbp2*/3, *Stat1*, and *Cxcl1*) and extracellular matrix remodeling (e.g., *Itga2*); key genes related to beta cell identity or insulin secretion (e.g., *Ucn3*, *MafA*, and *Ins1*) and metabolic regulation (e.g., *Fh1*) were decreased (Fig. 4, *C* and *D*). Interestingly, genes and pathways that were upregulated tended to be more upregulated in 35 versus 55 mg/kg group, and the genes that were downregulated were more downregulated in 55 versus 35 mg/kg group, suggesting a dose-dependent effect of STZ on gene expression. There was a small subset of genes that were altered in opposing directions with the different STZ doses: 29 genes were down in 35 mg/kg group and up in 55 mg/kg group (e.g., *Ptma*, *Batf2*, *Rhbdd3*, and *Rmi1*) and 8 genes were up in 35 mg/kg group and down in 55 mg/kg group (e.g., *Lars2*, *Zfp612*, *Map1b*, and *Thoc2*) (Fig. 4, *B* and *D*).

### STZ Dose-Dependent Differential Expression of Genes and Pathways Related to Endocrine Cell Function and Survival

**Figure 5.**
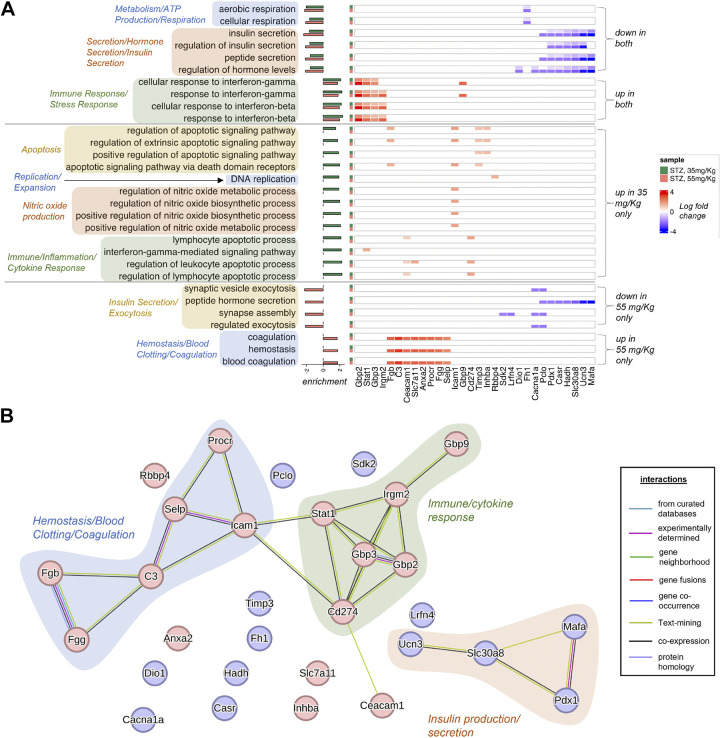
Pathway analysis of 35 mg/kg STZ vs. 55 mg/kg STZ. *A*: heatmap of top differentially expressed genes (|log fold-change| > 1.5) among the enriched biological processes in STZ, 35 and 55 mg/kg samples, grouped process type. For each process, the log fold-changes in both samples are shown, color coded on the left according to sample type (35 mg/kg: green and 55 mg/kg: red). Bar plot on the left reflects normalized enrichment scores (NESs) as obtained from gene set enrichment analysis. *B*: protein interaction network from STRING database involving the genes highlighted in *A*. Subnetworks related to major enriched processes are highlighted in different shades. Up and down genes are colored red and blue, respectively. STZ, streptozotocin.

Despite the overall similarities in differentially expressed genes (Fig. 4), the 35 and 55 mg/kg animals had distinct patterns of gene expression. In both groups, there were similar patterns of differentially expressed genes involved in pathways related to metabolism/ATP production/respiration, insulin/hormone secretion, and immune/stress response. Notably, there were dose-dependent differences in the degree to which these pathways were disrupted (Fig. 5, *A* and *B*).

We characterized the pathways between 35 mg/kg- and 55 mg/kg-treated animals that were differentially expressed and found that compared with the 55 mg/kg-treated animals, 35 mg/kg-treated animals had increased expression of genes related to pathways involving apoptosis, replication/expansion, nitric oxide production, and inflammatory/cytokine response. Alternatively, 55 mg/kg had downregulation of insulin exocytosis pathways not observed in 35 mg/kg as well as increased expression of pathways related to hemostasis/blood clotting/coagulation (Fig. 5*A*). Changes in the expression of genes in these pathways support evidence of a greater loss of insulin secretion in 55 mg/kg-treated mice compared with 35 mg/kg-treated mice observed in hyperglycemic clamps (Fig. 3).

## DISCUSSION

Rodent models of diabetes such as STZ-induced diabetes are essential tools to examine the progression of functional beta cell mass loss in diabetes and are commonly used to evaluate strategies to prevent or reverse beta cell loss (4). Administration of multiple low doses of STZ highlights many features of human T1D progression such as immune infiltration, beta cell dysfunction, regeneration, and repair (5–9); this experimental model allows researchers to evaluate strategies to prevent or reverse beta cell loss. Despite the widespread use of multiple low-dose STZ-induced models of diabetes, there remains little uniformity in the dose used. In addition, there are still uncertainties regarding the mechanisms driving disease progression in STZ-induced models of diabetes (2, 3). We demonstrate here that STZ dose is a critically important factor to the onset and progression of STZ-induced diabetes models. We identified significant differences in disease progression between 35 and 55 mg/kg STZ doses. Given the widespread issues with reproducibility of animal models in scientific research (35–37), researchers should, therefore, be aware of the impact of a chosen STZ dose and carefully select and report the dose used.

We discovered that despite similar levels of beta cell loss, 35 mg/kg-treated animals remained glucose tolerant for significantly longer than 55 mg/kg-treated littermates. We posited that the 35 mg/kg-treated animals may have a longer period of sustained beta cell function. To test this hypothesis, we performed hyperglycemic clamps in STZ-treated mice, which we believe is the first time such experiments have been reported. Data supported our hypothesis and demonstrated that 4 days following STZ administration, 35 mg/kg-treated mice secreted significantly more insulin than 55 mg/kg-treated mice and, therefore, were able to better manage hyperglycemia despite having similar levels of beta cell mass loss. Thus, the two doses allow one to uncouple beta cell loss from function.

From the hyperglycemic clamps, we were able to distinguish between first and second phases of insulin secretion in both 35 mg/kg STZ- and 55 mg/kg STZ-treated groups. This aspect of our study is critical, given that loss of first-phase insulin secretion, which is defined as the insulin secreted in the first 10 min following an increase in blood glucose, is an important hallmark of diabetes onset and represents a clinically important indicator of beta cell dysfunction (38). We determined that although there was a decrease in peak insulin secretion in 35 mg/kg-treated mice, the total AUC for 35 mg/kg-treated mice was not significantly lower than saline controls. Both 35 mg/kg STZ- and 55 mg/kg STZ-treated mice did, however, have a loss of second-phase insulin secretion compared with saline-treated control mice. Although mechanistically distinct from the first phase, second-phase insulin secretion is physiologically significant in that it can be sustained for hours after hyperglycemia is induced, accounts for the majority of insulin secretion following a meal, and is a major regulator of glucose homeostasis. Additional work should be done to fully describe the mechanisms impacting both first- and second-phase insulin secretion following multiple low-dose STZ.

To elucidate the molecular mechanisms governing disease progression in the STZ-treated mice, we assessed the impact of STZ dose on gene expression from isolated islets. We concluded that the changes in gene expression were generally similar between the doses and aligned with previously published transcriptomic reports (34, 39). However, we discovered significant dose-dependent changes in gene expression that had not previously been reported. The higher dose of STZ induced changes in the expression of nearly five times more genes than 35 mg/kg STZ. Many of the common differentially expressed genes between the two doses are known to be associated with diabetes. These include genes related to beta cell identity and function (e.g., *Ins1*, *MafA*, *Ucn3*, and *Ppp1r1a*) or beta response to stress or inflammation (e.g., *Gbp2*/3, *Stat1*, and *Cxcl1*). Other differentially expressed genes include prospective therapeutic targets to treat diabetes such as MAP3K15 (40), as well as genes known to be associated with certain increased genetic risks for diabetes in humans (e.g., *Pcsk2* and *Pclo*) (41, 42).

Within the similarly disrupted pathways, we found that the 55 mg/kg STZ-treated mice tended to have a greater downregulation of transcriptionally suppressed pathways, whereas 35 mg/kg STZ-treated animals tended to have a greater upregulation of induced pathways. Comparing the doses revealed that several key pathways were in fact differentially expressed. The 35 mg/kg-treated animals had relatively increased expression of genes in pathways regulating replication/expansion, nitric oxide production, apoptosis, and inflammatory/cytokine response (e.g., *Icam1*, *Rbbp4*, and *Cd274*). Conversely, 55 mg/kg-treated animals had decreased expression of beta cell identity- and insulin secretion/exocytosis-related genes (e.g., *Pdx1*, *Ucn3*, *MafA*, and *Slc30a8*).

We acknowledge that both the 35 and 55 mg/kg doses can be valuable tools for researchers. A 55 mg/kg dose reliably induces a substantial but incomplete loss of functional beta cell mass to induce glucose intolerance in a relatively short period (less than 3 days). Alternatively, a lower dose of 35 mg/kg provides investigators with an extended period to study beta cell dysfunction and may therefore be a more appropriate model to test interventions intended to preserve or reverse functional beta cell mass loss, especially for those strategies requiring some time to develop. In addition, the 35 mg/kg dose may represent, at least in mice, a time period analogous to T1D diagnosis; on the other hand, the 55 mg/kg dose would align more closely with the weeks and months after diagnosis. Given the recent success of the first approval for a treatment that prolongs C-peptide levels in newly diagnosed people with T1D (43, 44), and presumably more to come (45, 46), more well-defined preclinical models can facilitate efforts to develop more early intervention therapies.

We do note that our findings have several limitations resulting from the animals we studied. All experiments were restricted to male C57BL/6J mice that were around 10 wk old. Male mice are preferred to female mice, which are known to be largely resistant to STZ-induced diabetes and have significant differences in how they respond to STZ (47–49). Likewise, different strains of mice have been documented to respond to STZ in significantly different ways with some requiring significantly larger doses to induce diabetes (47, 50–52). In addition, beta cell dysfunction (53), as well as the efficiency of STZ-induced beta cell damage, is known to be age dependent (54). However, these findings can be a stepping stone to understanding the importance of dose and future studies can, and already have begun to, elucidate sex- and strain-specific differences in the response to STZ.

We conclude that variations in multiple low-dose STZ regimens can elicit significant differences in the progression of functional beta cell mass loss in mice. Although a range of doses are used to induced diabetes, dose selection has significant impacts on the rate at which beta cell function declines. Although beta cell mass progression appeared to decline similarly between 35 and 55 mg/kg doses, the loss of beta cell function progressed significantly faster in 55 mg/kg-treated mice compared with 35 mg/kg-treated mice. The higher dose of STZ also elicited more robust changes in gene expression. The prolonged development of diabetes with the lower dose of STZ could be advantageous to testing the efficacy of emerging therapies in preclinical models. Thus, researchers should take careful precautions in selecting, preparing, and reporting STZ doses when using STZ-induced models of diabetes.

## DATA AVAILABILITY

Data will be made available upon reasonable request.

## SUPPLEMENTAL DATA

10.5281/zenodo.8045137 Supplemental Table S1: https://doi.org/10.5281/zenodo.8045137.

## GRANTS

This research was funded by National Institutes of Health Grant DK099311 (to P.T.F.) and Innovation Awards from the Wanek Family Project to Cure Type 1 Diabetes (to P.T.F.). This work utilized services from the Center for Comparative Medicine and Solid Tumor Pathology, Integrated Genomics, and Light Microscopy Cores at the City of Hope, which were supported by the National Cancer Institute of the National Institutes of Health Grant P30CA033572.

## DISCLOSURES

No conflicts of interest, financial or otherwise, are declared by the authors.

## AUTHOR CONTRIBUTIONS

B.M.B. and P.T.F. conceived and designed research; B.M.B., E.B.-S., and J.M.I.-D. performed experiments; B.M.B., S.B., E.B.-S., J.M.I.-D., and P.T.F. analyzed data; B.M.B., S.B., J.M.I.-D., and P.T.F. interpreted results of experiments; B.M.B. and S.B. prepared figures; B.M.B., E.B.-S., and P.T.F. drafted manuscript; B.M.B., S.B., E.B., J.M.I.-D., and P.T.F. edited and revised manuscript; B.M.B., S.B., E.B.-S., and P.T.F. approved final version of manuscript.
